# Crystal structure modification of nano-hydroxyapatite using organic modifiers and hydrothermal technique

**DOI:** 10.1039/d4ra03111c

**Published:** 2024-09-18

**Authors:** Md. Kawsar, Md. Sahadat Hossain, Sumaya Tabassum, Dipa Islam, Newaz Mohammed Bahadur, Samina Ahmed

**Affiliations:** a Institute of Glass & Ceramic Research and Testing, Bangladesh Council of Scientific and Industrial Research (BCSIR) Dhaka-1205 Bangladesh shanta_samina@yahoo.com; b Department of Applied Chemistry and Chemical Engineering, Noakhali Science and Technology University Noakhali Bangladesh; c Biomedical and Toxicological Research Institute (BTRI), Bangladesh Council of Scientific and Industrial Research (BCSIR) Dhaka-1205 Bangladesh

## Abstract

Hydroxyapatite (HAp) synthesis was achieved through a hydrothermal method involving orthophosphoric acid and calcium hydroxide. Different organic modifiers such as urea, naphthalene, and palmitic acid were applied in the reaction system to modify the crystallite size along with the morphology of HAp. The synthesized HAp was validated *via* X-ray diffraction (XRD) data, Fourier Transform Infrared (FTIR) spectra, Field Emission Scanning Electron Microscopy (FESEM) image, and optical bandgap energy (<6 eV) was determined through UV-vis spectrophotometry. Apart from that, different techniques such as Scherrer's method, Halder–Wagner model, Williamson–Hall method, size–strain plot, as well as Sahadat-Scherrrer's models were applied for calculating the crystal domain size, and some models also incorporated energy density, strain, and stress. The synthesized HAp has a crystal structure that falls within the permissible range of <100 nm, as established by analyzing the XRD data using established models. Nevertheless, the values for strain (from −0.0006 to 0.0062), stress (from −30 902 to 36 940 N m^−2^), as well as energy density (from 4 × 10^−14^ to 113.72 J m^−3^) were likewise computed for the synthesized HAp. The texture co-efficient analysis reveals that doped HAp is grown in the (202) and (112) planes, palmitic acid_HAp in (002), (112), and (202) planes, while all the synthesized HAp (pure HAp, urea, naphthalene) is grown in the (002) and (112) planes. Rietveld refinement was also performed to estimate the quantative phase percentage from XRD data.

## Abstract 2

Hydrothermal technique and organic modifiers are effective tools for the modification of the crystal structure of nano-crystallite hydroxyapatite for fruitful applications.

## Graphical abstract

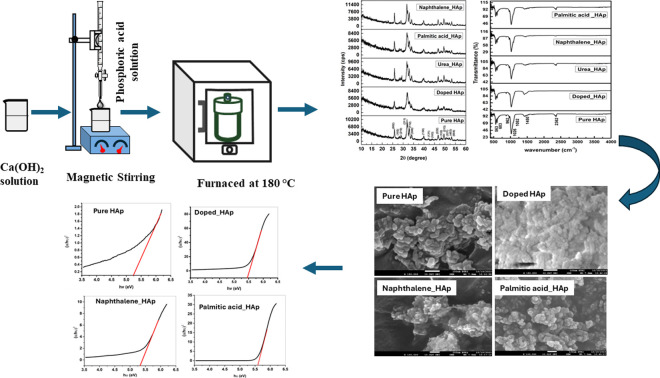

## Introduction

Hydroxyapatite (HAp) is a naturally occurring phosphate of calcium found in hard tissues, comprising 70% of tooth dentin, 60% of bones, and 97% of tooth enamel.^1,2^ Researchers are exploring synthetic biomaterials to repair or replace damaged organs or bones, addressing the struggles in the medical field to find suitable materials with appropriate bioactivity, biocompatibility, and physiological properties. They are exploring the fruitful applications of HAp in biomedical sectors as well as environmental fields, altering raw materials such as calcium and phosphate sources.^3^ The characteristics of HAp, such as morphology as well as size, significantly influence its applications. For instance, HAp whiskers can serve as reinforcement phases, enhancing the mechanical characteristic and reliability of HAp ceramics.^4^ HAp can be synthesized by numerous methods, including (1) dry methods such as mechanochemical^4^ and solid-state^5^ and (2) wet methods such as chemical precipitation,^6^ emulsion,^7^ sol–gel,^8^ hydrolysis, as well as the hydrothermal method.^9^ However, all these synthesis approaches have typical difficulties such as agglomeration, extended reaction periods, unpredictable particle size, as well as non-stoichiometric substances.^10^ Despite the multiplicity of synthesis techniques, the hydrothermal approach provides a unique method of influencing the crystal shape by applying high temperature and pressure.^11^ Numerous studies have utilized hydrothermal methods to synthesize one-dimensional HAp, including platelets, tubes, whiskers, and ribbons, with some reports incorporating organic modifiers,^12^ offering enormous benefits and additional advantages. This research addresses the potential of organic modifiers as well as hydrothermal methods for forming hydroxyapatite nanostructures, a strategy that differs from prior literature that focuses on the hydrothermal technique alone. Promoting the hydrothermal synthesis of HAp involves using urea, palmitic acid, and naphthalene as organic modifiers. Urea acts as a mineralizer, promoting nanorods and nanobundles. Palmitic acid, a surfactant, limits the growth and size distribution by adsorbing onto HAp crystals. Naphthalene forms pore-forming structures with high surface area and volume. Decomposition during hydrothermal processes may increase HAp's biological activity and drug transport capabilities.^13,14^ The initial stage of HAp formation involves a complex mechanism, with amorphous calcium phosphate formed as a transient phase using orthophosphate acid/salt as the PO_4_^3−^ precursor.

ACP is then subsequently converted into several phases, including HAp, octacalcium phosphate (OCP), tricalcium phosphate (TCP), as well as dibasic calcium phosphate dihydrate (DCPD). In this present study, HAp nanocrystal was synthesized employing the hydrothermal method along with different organic modifiers such as urea, naphthalene, and palmitic acid to modify the crystallite size as well as the morphology of HAp. Furthermore, crystallite size computation is achieved using various equations and models, including Scherrer's model, size–strain plot, Halder–Wagner model, Sahadat-Scherrer's model, and Williamson–Hall plot.^15^ These methods are easy to apply and can predict strain, energy density, and stress from the XRD data, making it a valuable tool for analyzing crystallite size. The synthesized HAp was also characterized through scanning electron microscopy (SEM), Fourier transform infrared spectroscopy (FTIR) as well as UV-vis-spectroscopy.

## Materials and methods

### Materials

Calcium hydroxide (Ca (OH)_2_) (96%), along with phosphoric acid (H_3_PO_4_) (85%), were employed as potential sources for Ca^2+^ and PO_4_^3−^, respectively, to execute this experiment. Additionally, numerous organic modifiers such as urea (CH_4_N_2_O) (99%), naphthalene (C_10_H_8_) (99%), palmitic acid (C_16_H_32_O_2_) (98%), as well as externally synthesized HAp (C_10_(PO_4_)_6_(OH)_2_) were employed to modify the morphology of the synthesized HAp nanocrystals. These chemical reagents were purchased from E-Merck, Germany. Apart from that, nitric acid (HNO_3_) and ammonium hydroxide (NH_4_OH) were utilized to maintain the pH (10–11). DI water was used as a solvent throughout the whole synthesis process.

## Method

### Synthesis of nano HAp *via* the hydrothermal technique

**Fig. 1:**
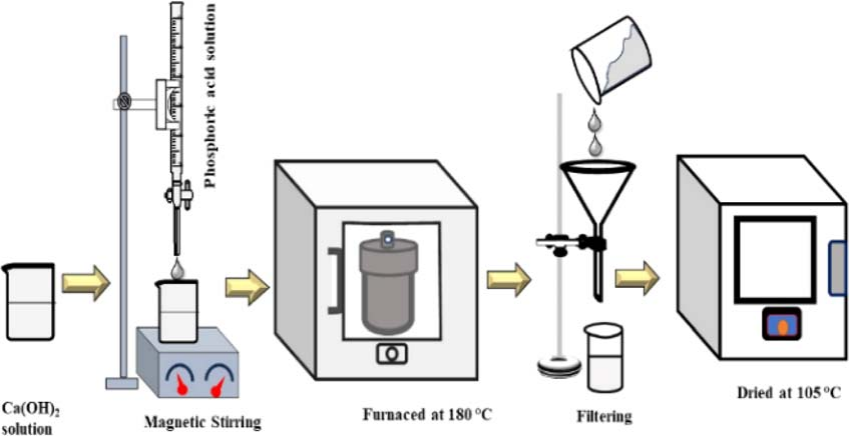
Experimental procedure for hydrothermal HAp synthesis.

The synthesis process of HAp nanocrystals using the hydrothermal technique is shown in Fig. 1. Firstly, a predetermined amount of Ca(OH)_2_ was dissolved in DI water, then H_3_PO_4_ was added into the Ca^2+^-containing solution from the burette to maintain a molar ratio of (Ca/P) = 10 : 6. The addition of H_3_PO_4_ into this solution was done at the rate of 4 mL per min. During the synthesis process, the pH of the solution was maintained above 9 using NH_4_OH and/or HNO_3_ solution. Different organic modifiers such as urea (0.1 g), naphthalene (0.1 g), palmitic acid (0.1 g), as well as external HAp (0.1 g) were added into the reaction system, and the solution was stirred for 2 h at room temperature. Then, the solution was shifted into a 100 mL stainless-steel autoclave reactor encased in polytetrafluoroethylene (PTFE) and subjected to hydrothermal synthesis at a temperature of 180 °C for 3 h. Afterward, the resulting mixture was cooled to the ambient temperature. The precipitate was separated by passing it through a filter paper and then rinsed with deionized water. These precipitates were then subjected to the process of drying in an oven at 105 °C for 6 h.

## Characterization

### X-ray crystallographic characterization

A Rigaku SE X-ray diffractometer was used to validate the crystalline shapes of HAp nanocrystals with the scanning range of 2*θ* = 10–60° and scanning step of 0.01. The radiation source, CuKα (*λ* = 1.54060 Å), was employed at 40 kV as well as 30 mA, with cooling temperature of 19–20 °C. The observed phases were compared to the standard JCPDS data file.

### Functional groups analysis (FT-IR)

Synthetic gypsum was analyzed using an IR-Prestige 21 instrument, which had an IR spectral resolution of 4 cm^−1^ and operated in the transmittance mode within the 400–4000 cm^−1^ range. The analysis included 30 scans and used an attenuated total reflection (ATR) accessory.

### Surface morphology analysis (SEM)

The surface morphology of HAp was explored employing a scanning electron microscope (model: Phenom pro), with gold sputter-coated for 30 seconds and pictures acquired at 10 kV, including powder gypsum samples suspended in ethanol.

### UV-visible spectroscopic analysis

A UV-visible spectrophotometer (Hitachi U-2910) was employed to measure absorbance at 190–350 nm wavelengths, with tungsten as well as deuterium lamps utilized for UV-visible irradiation.

## Results and discussion

### XRD data interpretation

**Fig. 2:**
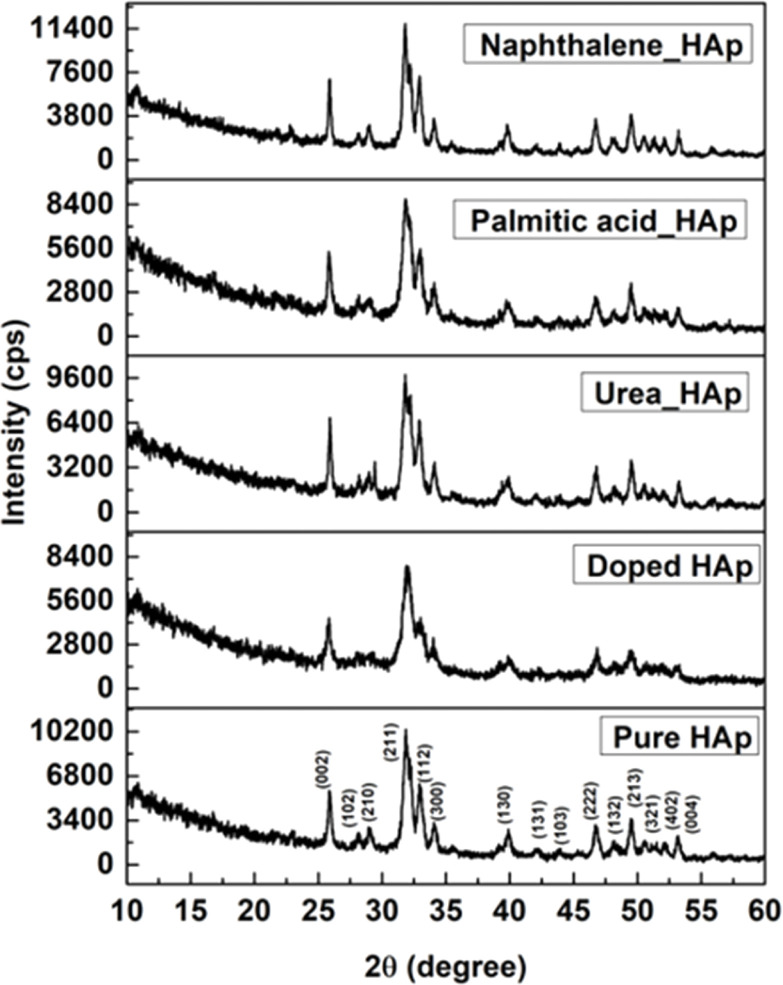
X-ray diffractogram of the synthesized HAp utilizing the hydrothermal technique.

The patterning results from XRD for the synthesized HAp through the hydrothermal technique are unveiled in Fig. 2. The crystallographic plane positions of these synthesized HAp were visible at (002) 25.92°, (211) 31.83°, 32.24°, (300) 32.96°, (202) 34.12°, (130) 39.88°, (222) 46.75°, and (213) 49.53°. The HAp profiles were matched by the standard ICDD database (card no: #01-074-0565). From the analyzed data, several crystallographic characteristics such as lattice parameters, crystalline index, dislocation density, unit cell volume, crystallite dimensions, microstrain, as well as the degree of crystallinity were estimated utilizing eqn (1)–(7), as well as summarized in Table 1.^16–18^1
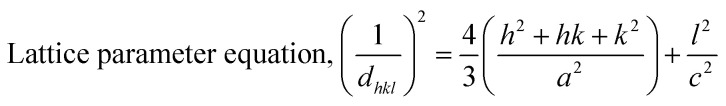
2
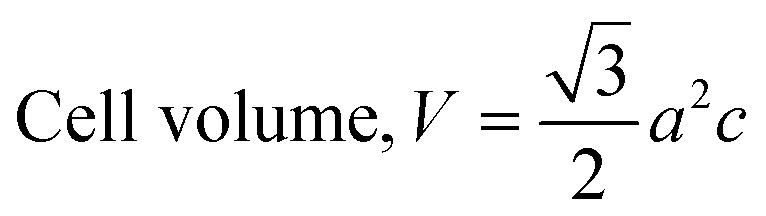
3
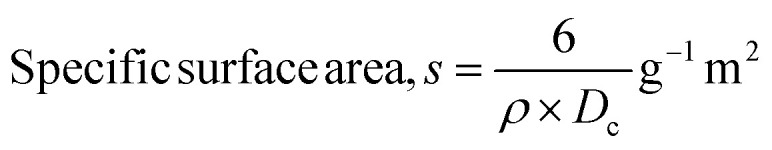

4 Microstrain, *ε* = *β*/4 tan *θ*

5
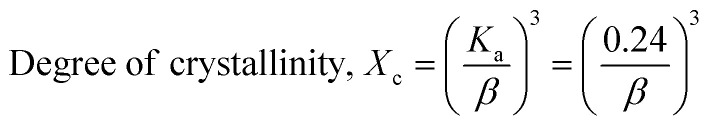
6
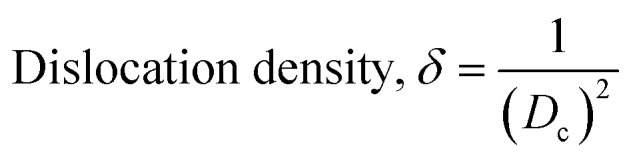
7
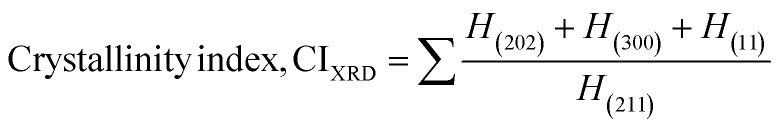
here, the unit cell is designated by plane (*h*, *k*, and *l*), whilst lattice parameters are represented by *a*, *b*, *c*. In addition, the degree of crystallinity is defined as *X*_c_.^19–21^ Dislocation density, the peak height of the respective plane, and crystalline index are denoted by *δ*, *H*_(*hkl*)_, CI_XRD_ respectively. By implementing eqn (3), the specific surface area of the prepared HAp was calculated using the size of the crystallite and specific density, noted as *D*_c_ and *ρ* (3.16 g cm^−3^), respectively.^22^ The polycrystalline material's preferred orientation is defined by its texture coefficient, obtained from its X-ray diffraction pattern, which defines the variation in the material's crystallographic planes.^23^

**Table 1:** Crystallographic characteristics of hydrothermally produced HAp

Parameter	Pure_HAp	Doped_HAp	Urea_HAp	Naphthalene_HAp	Palmitic acid_HAp
Lattice parameter (Å)	*a* = *b* = 9.46	*a* = *b* = 9.46	*a* = *b* = 9.41	*a* = *b* = 9.42	*a* = *b* = 9.42
	*c* = 6.88	*c* = 6.88	*c* = 6.88	*c* = 6.88	*c* = 6.89
Volume of unit cell (Å^3^)	*V* = 534.21	534.79	528.16	529.62	529.94
Degree of crystallinity	*X*_c_ = 0.035	1.92	0.154	0.308	0.027
Microstrain	*ε* = 11.14 × 10^−3^	3.014 × 10^−3^	6.852 × 10^−3^	5.444 × 10^−3^	12.12 × 10^−3^
Dislocation density, (10^15^ lines per m^2^)	*δ* = 7.787	0.567	2.928	1.847	9.215
Crystallinity index	CI_XRD_ = 1.446	1.926	1.784	1.451	1.633
Specific surface area	*S* = 0.1675	0.0452	0.10275	0.08160	0.1822

The texture coefficient, computed by comparing a particular peak in an XRD pattern to a reference peak, gives essential information about a material's crystallographic orientation, which may greatly affect its qualities and performance in numerous applications. Eqn (8) can be applied to calculate the texture coefficient of the synthesized HAp.8
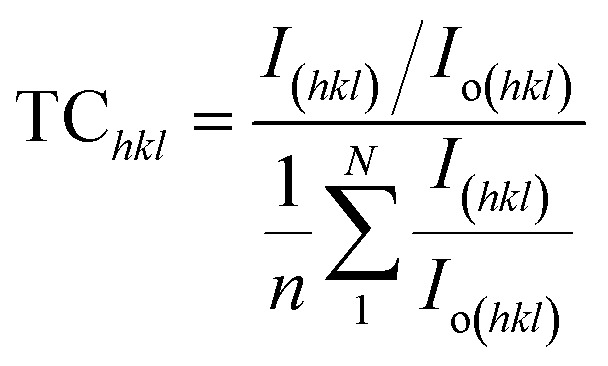
here, the term *I*_(*hkl*)_/*I*_o(*hkl*)_ is the ratio of the sample relative intensity concerning the standard relative intensity and 
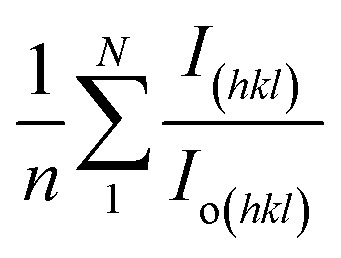
; it is the average of relative intensity, correspondingly. The analysis shows that the texture co-efficient for hydrothermally synthesized pure HAp, urea_HAp, and naphthalene_HAp is grown in the (002) and (112) planes. Conversely, doped_HAp is grown in the (202) and (112) planes, whereas palmitic acid_HAp is grown in the (002), (112), and (202) planes, respectively.

### Estimation of crystallite size using XRD models

Numerous XRD models were utilized along with Scherrer's model to estimate the crystallite dimension precisely. Additionally, versatile applicable methods such as Williamson–Hall, Halder–Wagner, and Sahadat-Scherrer are used.

### Scherrer's model

Scherrer's model is the first model that showed the relationship between crystallite size (eqn (8)) and peak broadening.^24,25^ XRD diffraction patterns widen in the nanocrystal because of the intrinsic and crystalline size effects, which can be distinguished into two parts: instrumental broadening and physical broadening.^26–28^ In the following equation, the instrumental as well as crystalline size effects are not considered.9
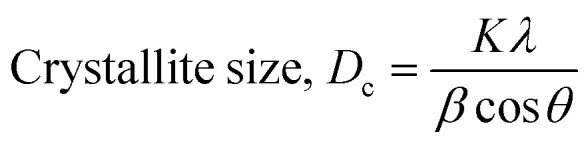
here, *D*_c_ is identified as the size of a unit cell. Consequently, FWHM (full width at half maxima) in radian is specified as *β*. Shape factor or Scherrer's constant, as well as the degree of crystallinity, are identified as *K* = 0.90. The average crystalline size of the synthesized HAp was found to be 20 nm, 12 nm, 25 nm, 30 nm, and 25 nm for Pure HAp, Doped HAp, Urea HAp, Naphthalene HAp, as well as Palmitic acid Hap, respectively.

### Williamson–Hall method

In Scherrer's method, just the impact of crystalline size on the XRD peak widening is considered, but it does not provide any indication regarding the microstructure of the lattice, which can be generated because of point defect, triple junction, stacking faults as well as grain boundary.^29–31^ According to this method, the sum of line broadening in the XRD diffraction pattern can be written as

10 *β*_total_ = *β*_size_ + *β*_strain_

The slide modification in the Williamson–Hall, regarded as the UDM, USDM, and UDEDM, will be investigated in this context, where *β*_strain_ is denoted as the broadening due to strain while *β*_size_ is the widening owing to crystallite dimension.^32^

### Uniform deformation model (UDM)

In the UDM, homogenous strain is possessed through crystallographic planes, which appears to be due to crystal imperfections. More specifically, UDM deals with isotropic strain in crystallographic analysis.^33,34^ The mathematical estimation of the physical widening of the XRD peaks caused by intrinsic strain effects may be determined as

11 *β*_strain_ = 4*ε* tan *θ*

where *θ* indicates Bragg position, and the rearrangement of eqn (9)–(11) gives12
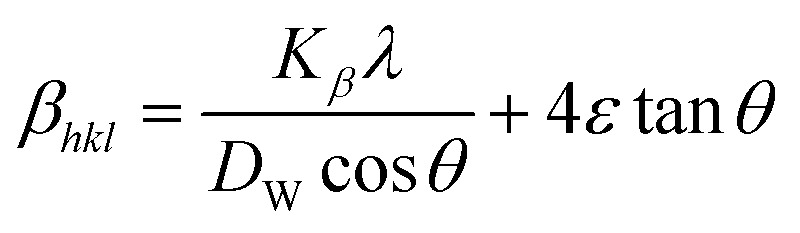
from altering eqn (12) it gives13
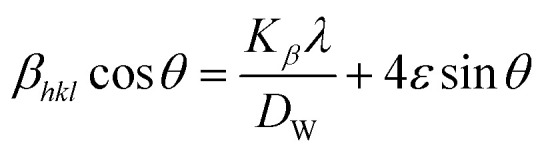


**Fig. 3:**
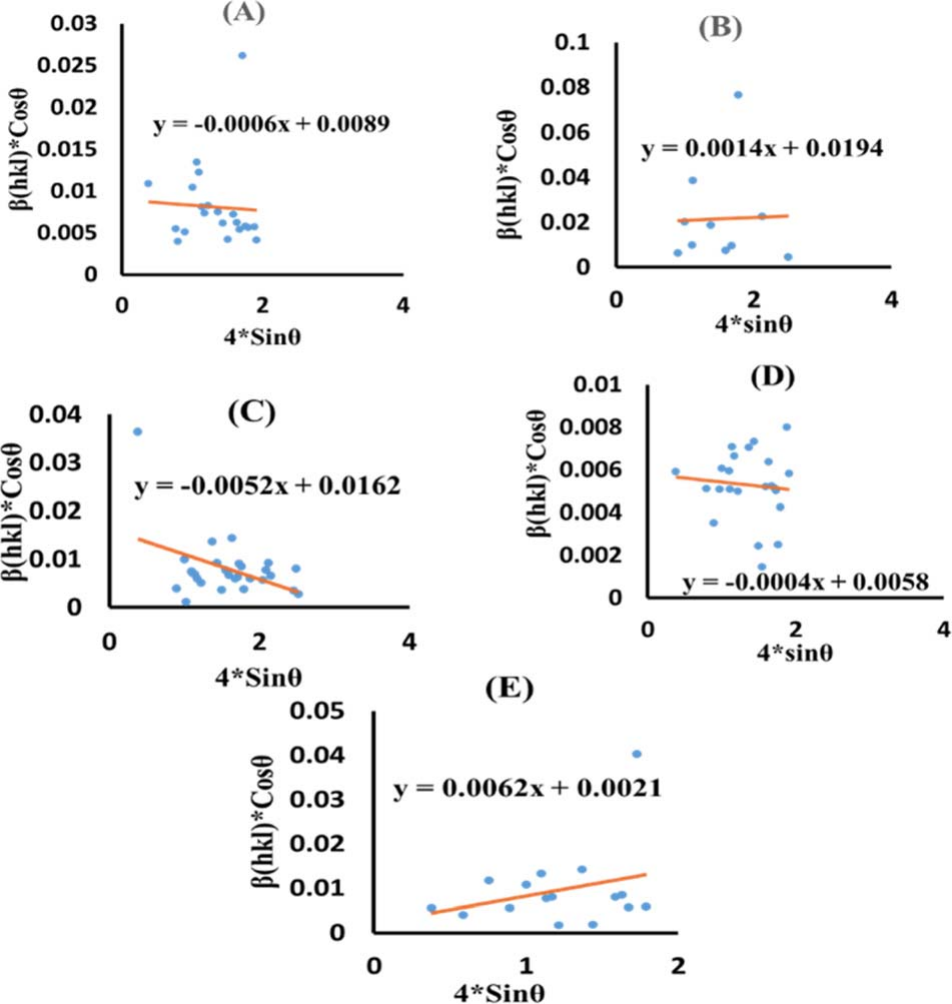
UDM plot for synthesized HAp nanoparticles. (A) Pure Hap, (B) Doped_Hap, (C) Urea_Hap, (D) Naphthalene_Hap, (E) Palmitic acid_HAp.

Eqn (13) is graphically illustrated in Fig. 3, with *β*_*hkl*_ *cos *θ* and 4 sin *θ* as independent variables along the vertical as well as the horizontal axis. The slope represents the strain in HAp nanocrystals, while the intercept shows the mean particle size due to lattice contraction or expansion. This modification slightly alters the atomic arrangement and creates defects in the lattice structure, resulting in strain. The average crystallite size was obtained at 15.57, 7.14, 8.55, 23.90, and 66.02 nm for Pure HAp, Doped_HAp, Urea_HAp, Naphthalene_HAp as well as Palmitic acid_Hap, respectively. The UDM plot shows a positive slope resulting in lattice expansion, intrinsic strain in nanocrystals, and a negative magnitude, indicating compressive strain.^35^

### Uniform stress deformation model (USDM)

The actual crystal, being anisotropic, does not match the UDM model's homogeneous and isotropic assumption. The USDM is a modified version of the Williamson–Hall equation, assuring uniform lattice deformation stress with a minimal microstrain.^28,36^ Hooke's law illustrates a linear connection between stress (*σ*) as well as strain (*ε*), as illustrated in eqn (14).

14 *σ* = *Y*_*hkl*_*ε*

The equation gives a valid estimate for minimum strain, proving that Young's modulus (*Y*_*hkl*_) is not linear as strain increases.^37^ The relationship can be obtained by rearranging and substituting eqn (14) with eqn (10), which gives eqn (15).15
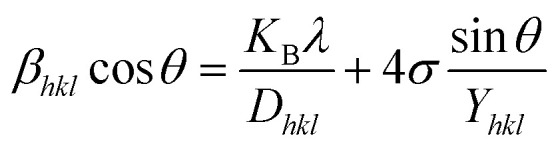


**Fig. 4:**
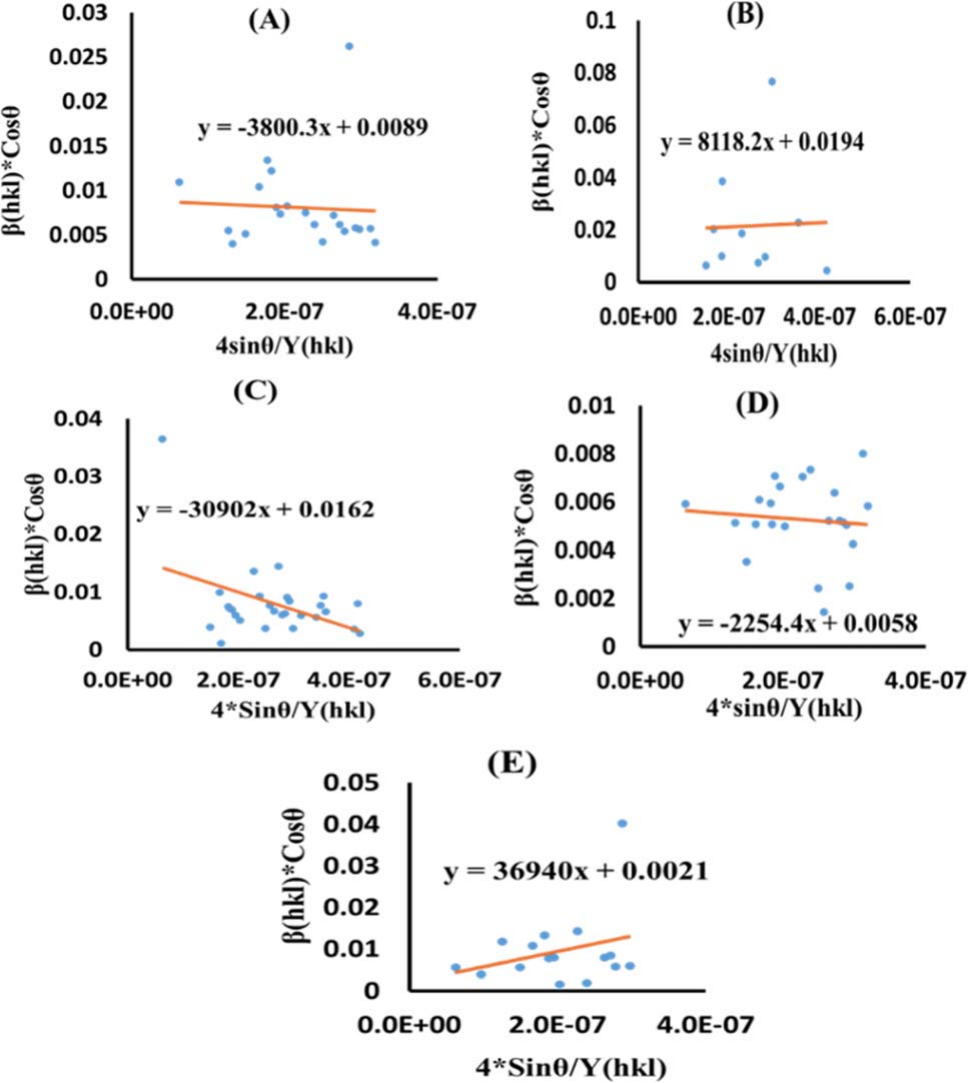
USDM plot for synthesized HAp nanoparticles. (A) Pure Hap, (B) Doped_Hap, (C) Urea_HAP, (D) Naphthalene_Hap, (E) Palmitic acid_Hap.

Now, the graphical representation of eqn (15), *β*_*hkl*_ *cos (*θ*) along the vertical as well as 4 sin *θ*/*Y*_*hkl*_ along the horizontal axis aligning to every diffraction pattern for synthesized HAp, has been visualized in Fig. 4. The crystallite dimension of the synthesized HAp was estimated by calculating *σ* from the linear fitting slope and extrapolation intercept. Table 2 provides the crystalline size and stress.

**Table 2:** Analysis of the microstructural properties of HAp *via* different models

XRD model name		Crystallite size (nm), stress (N m^−2^), energy density (J m^−3^)				
		Pure_HAp	Doped_HAp	Urea_HAp	Naphthalene_HAp	Palmitic acid_HAp
Scherrer's equation		*D*_L_ = 20.915	12.487	25.408	30.792	25.21
Williamson–Hall method	UDM	*ε* = −0.0006	*ε* = 0.0014	*ε* = −0.0052	*ε* = −0.0004	*ε* = 0.0062
		*D*_w_ = 15.57	*D*_w_ = 7.147	*D*_w_ = 8.558	*D*_w_ = 23.9058	*D*_w_ = 66.02
	USDM	*σ* = −3800.3	*σ* = 8118.2	*σ* = −30 902	*σ* = −2254.4	*σ* = 36 940
		*D*_w_ = 15.57	*D*_w_ = 7.147	*D*_w_ = 8.558	*D*_w_ = 23.9058	*D*_w_ = 66.02
	UDEM	*u* = 1.2036	*u* = 5.49	*u* = 79.577	*u* = 4 × 10^−14^	*u* = 113.72
		*D*_w_ = 15.57	*D*_w_ = 7.147	*D*_w_ = 8.558	*D*_w_ = 23.905	*D*_w_ = 66.02
Size–strain plot		*D*_w_ = 12.379	2.6562	3.7677	23.500	17.117
Halder–Wagner method		*D*_w_ = 5.43	3.3717	4.1493	13.5135	3.64
Sahadat-Scherrer's model		D_s–s_ = 23.5	17.55	43.329	39.615	43.32

### Uniform deformation energy density model (UDEDM)

The UDEDM is a novel model that effectively manages the homogenous anisotropic lattice strain across all crystallographic orientations, influenced by the deformation energy density. It differs from the USDM model, which considers the crystal's isotropic nature and is characterized by defects, dislocations, and agglomerates.^38^ In accordance with Hooke's law, energy density (*u*) corresponds to strain (*ε*), considering the relationship.16
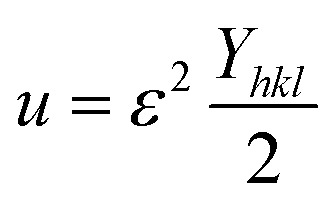


**Fig. 5:**
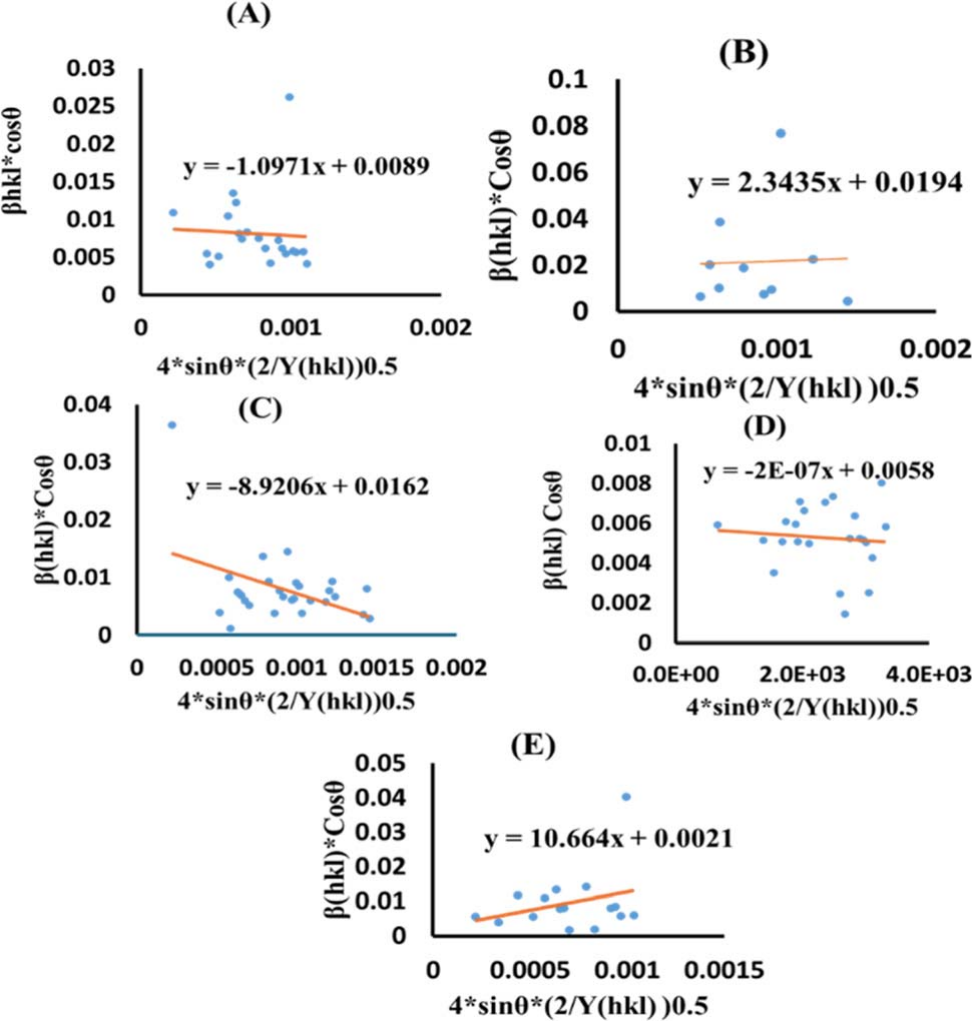
UDEDM plot for synthesized HAp nanoparticles. (A) Pure Hap, (B) Doped_Hap, (C) Urea_HAP, (D) Naphthalene_Hap, (E) Palmitic acid_Hap.

The UDEDM equation was constructed by restructuring eqn (16) regarding *ε*, then substituting it with eqn (13), resulting in eqn (17).17
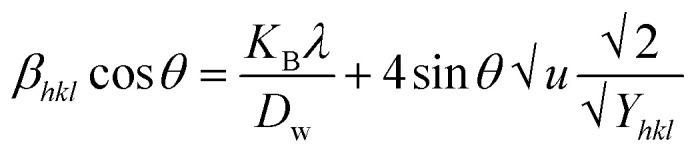
here, eqn (17) was plotted with the term *β*_total_ cos *θ* on the vertical axis and 
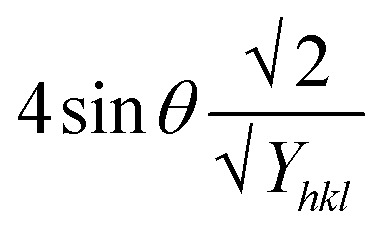
. On the horizontal axis (Fig. 5), the crystallite dimension, as well as the anisotropic density, were measured from the *Y*-intercept as well as the slope of the mentioned equation and is listed in Table 2.

### Size–strain plot (SSP)

Peak widening is influenced by size as well as strain components, with the component of size described by the Lorentzian function whereas the strain-induced component is defined by the Gaussian function.^32^ Consequently, the broadening of SSP can mathematically be estimated as follows.

18 *β*_total_ = *β*_L_ + *β*_G_

The peak widening owing to Lorentz and Gaussian functions is symbolized by *β*_L_ and *β*_G_, correspondingly.

Additionally, the SSP technique gives improved results for isotropic broadening owing to its concentration on low-angle reflections, assuring greater precision as well as accuracy. Higher angles result in lower-quality XRD data and significantly overlapped peaks, decreasing the accuracy of the results. Eqn (19) represents the SSP approach for the estimation of average strain as well as crystallite size.^28,39^19
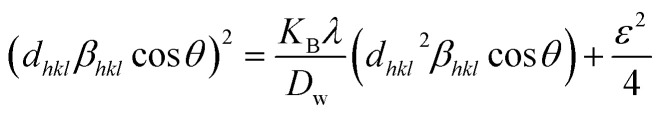


**Fig. 6:**
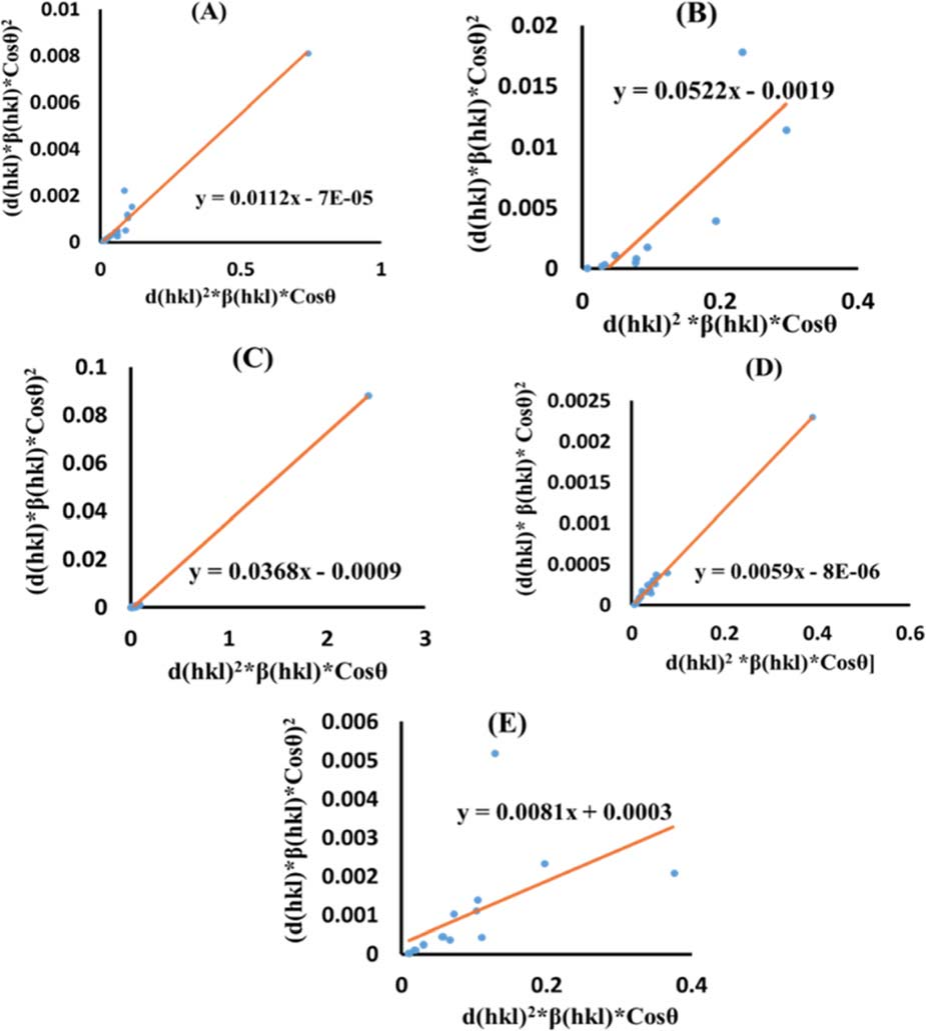
SSP for synthesized HAp nanoparticles. (A) Pure Hap, (B) Doped_Hap, (C) Urea_Hap, (D) Naphthalene_Hap, (E) Palmitic acid_Hap.

The lattice separation among the (*hkl*) planes is indicated by *d*_*hkl*_. The crystallite size was measured using a plot of (*d*_*hkl*_*β*_*hkl*_ cos *θ*)^2^ on the vertical axis and (*d*_*hkl*_^2^*β*_*hkl*_ cos *θ*) on the horizontal axis (Fig. 6). The crystallite size of the prepared samples was estimated as 12.37 nm for pure HAp, 2.65 nm for doped HAp, 3.76 nm for urea HAp, 23.5 nm for naphthalene HAp, and 17.11 nm for palmitic acid HAp.

### Halder–Wagner method (HWM)

The SSP technique considers strain widening as a Gaussian function and size broadening as a Lorentzian function. However, the XRD peak is either a Lorentzian or Gaussian function as its peak area fits the Gaussian function, but its tail goes too rapidly.^40^ The Halder–Wagner technique is employed to solve this issue, provided that peak broadening is a symmetric Voigt function.^41,42^ Hence, the Voigt function may be represented as the FWHM of the physical profile employing the H–W method.

20 *β*_*hkl*_^2^ = *β*_L_*β*_*hkl*_ + *β*_G_^2^

where *β*_G_, *β*_L_ = FWHM for Gaussian as well as Lorentzian function.21
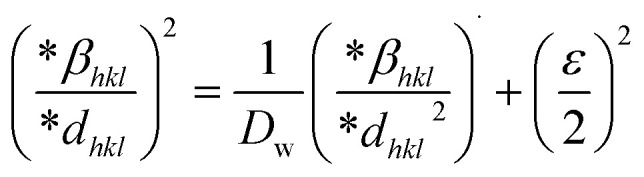
22
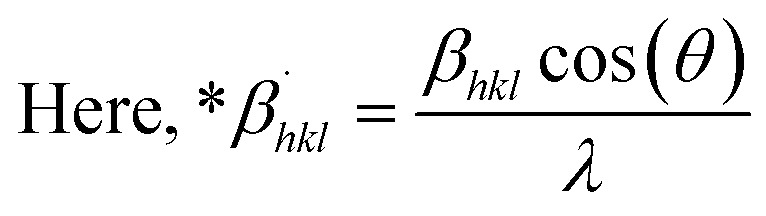
23
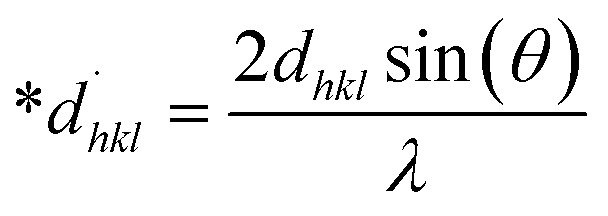


**Fig. 7:**
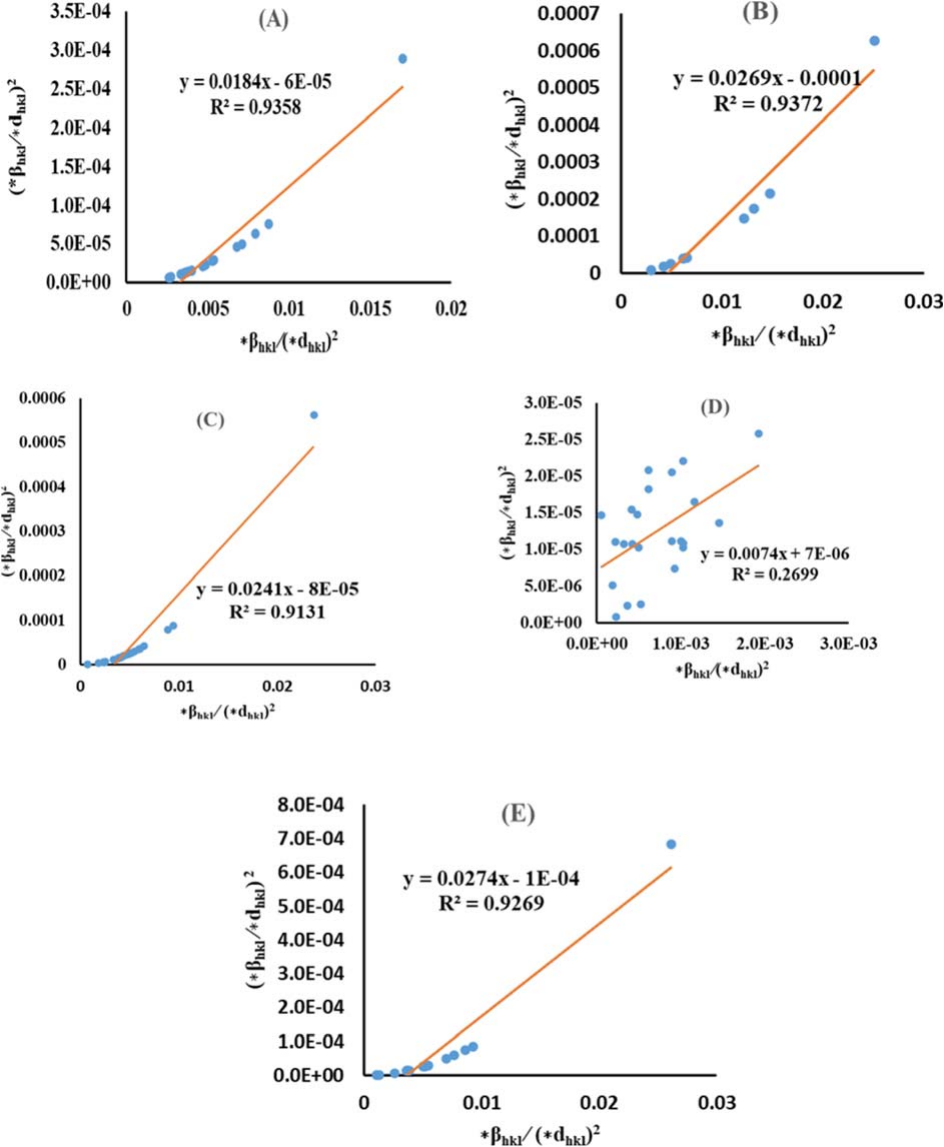
HWM for synthesized HAp nanoparticles. (A) Pure Hap, (B) Doped_Hap, (C) Urea_HAP, (D) Naphthalene_Hap, (E) Palmitic acid_Hap.

By plotting (*β_*hkl*_/*d_*hkl*_)^2^ as well as (*β_*hkl*_/*d_*hkl*_)^2^ upon the vertical as well as horizontal axis (Fig. 7), correspondingly. The crystallite dimension was estimated from the solp (1/*D*_w_) and is listed in Table 2. The crystallite size of the synthesized HAp was obtained as 5.43 nm for pure HAp, 1.93 nm for doped HAp, 4.14 nm for urea HAp, 13.51 nm for naphthalene HAp, and 3.64 nm for palmitic acid HAp.

### Sahadat-Scherrer model (SSM)

The SSM accurately estimates the crystallite size despite possible shortcomings in the prior models that might result in larger crystallite sizes.^43^ The method assessed each peak using a distinct linear trajectory passing through the origin, generating a more acceptable model for the direct line intersecting at the origin.^44,45^ The mathematical formula can be represented as eqn (24).24
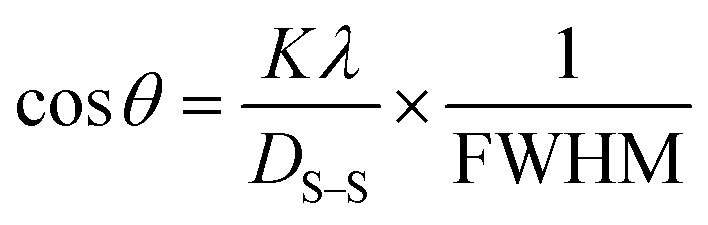


**Fig. 8:**
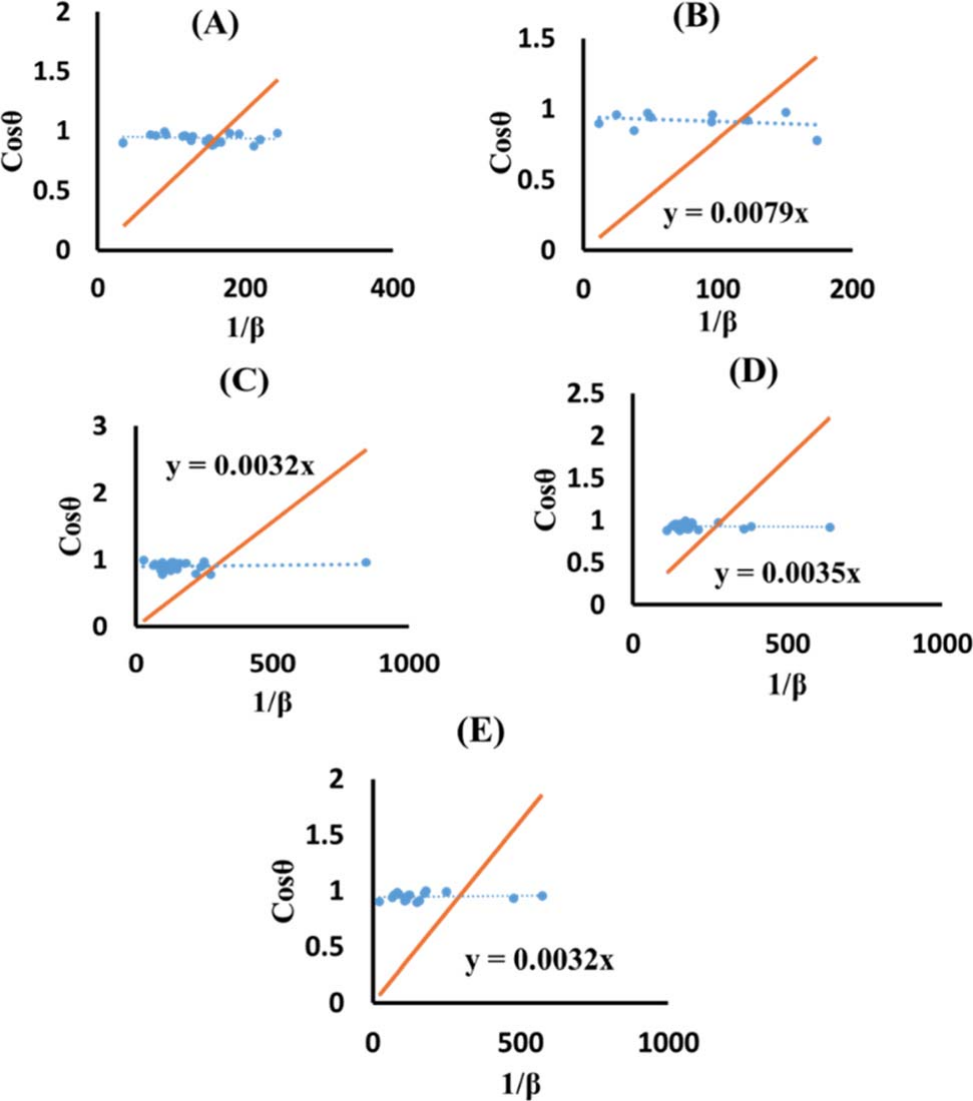
SSM for synthesized HAp nanoparticles. (A) Pure Hap, (B) Doped_Hap, (C) Urea_HAP, (D) Naphthalene_Hap, (E) Palmitic acid_Hap.

The terms 1/*β* and cos *θ* are plotted on the horizontal axis as well as the vertical axis (Fig. 8). An intercept was developed using Excel. The crystallite size was determined by calculating the slope of the linear equation, with pure, doped, urea, naphthalene, as well as palmitic acid HAp having a size of 23.5, 17.55, 43.32, 39.61 and 43.32 nm, respectively.

### Functional group analysis

**Fig. 9:**
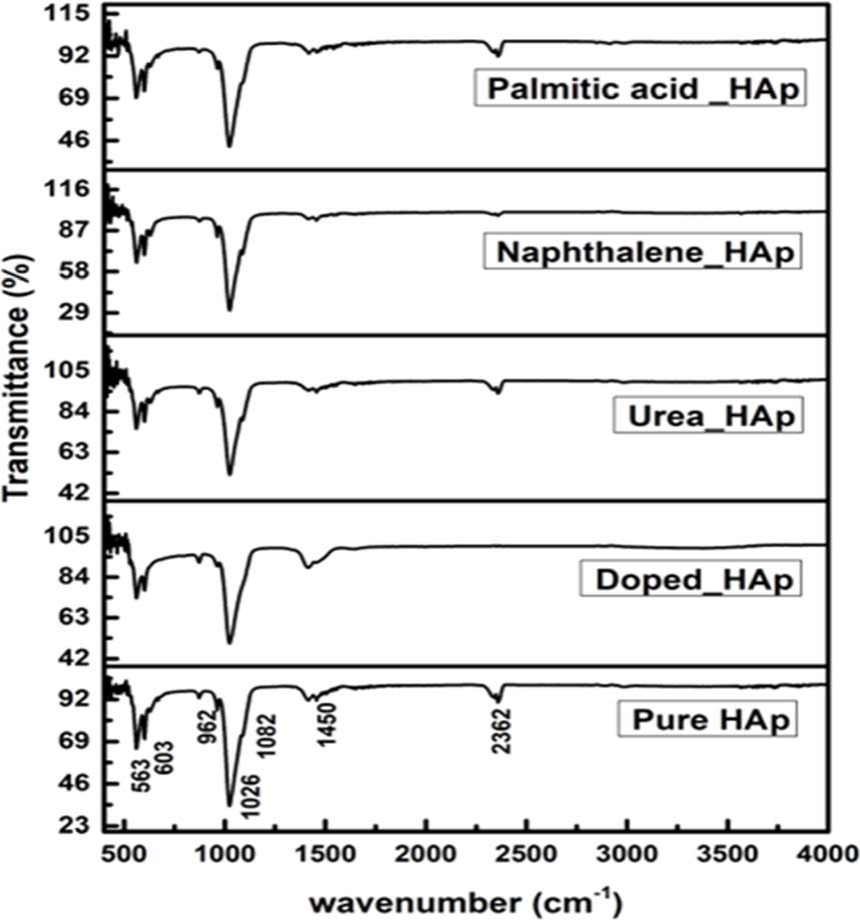
FTIR of hydrothermally synthesized HAp.

The observed spectra in Fig. 9 are attributed to the presence of PO_4_^3−^ as well as OH^−^ groups in HAp, as indicated by the FTIR spectra.^46^ The hydrothermal technique is used to alter the crystal structure of HAp, resulting in four early vibrations: symmetric stretching (*v*_1_), symmetric bending (*v*_3_), asymmetric stretching (*v*_2_), and asymmetric bending (*v*_4_).^47^ The study asserts that three types of stretching oscillations at 962, 1026, and 1087 cm^−1^ wavenumbers as well as the bending vibration peaks at the wavenumber near 465, 563, and 599 cm^−1^ are equivalent to HAp.^48,49^ The vibration at 962 cm^−1^ was ascribed to (*v*_1_) oscillation, whereas 1087 and 1026 cm^−1^ peaks were related to (*v*_2_) vibration. The vibrations (*v*_4_) are detected at wavenumbers 563 and 599 cm^−1^, whereas (*v*_3_) vibration is prevalent at 473 cm^−1^. Asymmetric bending vibration appears between 563 and 599 cm^−1^, while *v*_3_ is found at 473 cm^−1^. The FTIR peaks at 3000–3800 cm^−1^ wavenumber have been found in multiple publications, demonstrating comparable positions for OH^−^.^50,51^ The peak near 2350 cm^−1^ was due to the presence of environmental carbon dioxide.

### Assessment of optical properties

A UV-vis spectrophotometer was used to estimate the optical band gap of hydrothermally produced HAp at ambient temperature, requiring the Tauc plot technique to estimate absorption frequency (shown in eqn (25)).^52,53^

25 *αhθ* = *A*(*hθ* − *E*_g_)^*n*^

**Fig. 10:**
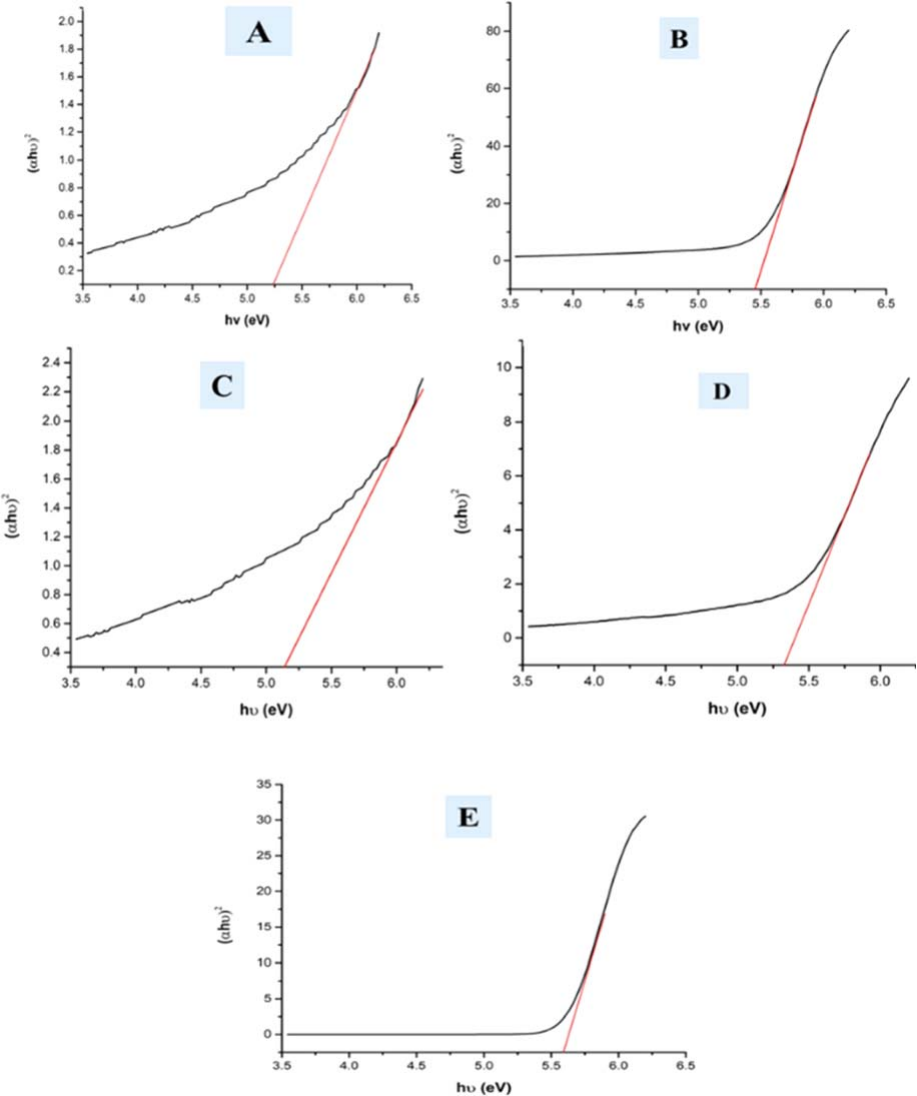
Optical band gap of (A) Pure Hap, (B) Doped_Hap, (C) Urea_HAP, (D) Naphthalene_Hap, (E) Palmitic acid_HAp.

In the mentioned eqn (25), *α*, *h*, and *θ* are identified as the adsorption coefficient, Planck's constant, as well as photon frequency. The data shows optical band gap (*E*_g_) values for various samples, including pure HAp, doped_HAp, Urea_Hap, Naphthalene_Hap, and Palmitic acid_HAp (Table 3). Disparities in the band gap may be due to dopants and organic modifications affecting the materials' electrical structure and optical characteristics (Fig. 10). Pure HAp has a band gap of 5.25 eV, while doped_HAp has a larger gap of 5.45 eV due to the dopant ions affecting the electrical structure. Urea_HAp has a reduced gap of 5.13 eV, possibly due to urea-derived functional groups or defects. Naphthalene_HAp has a band gap of 5.32 eV, lower than that of doped HAp but greater than that of pure HAp. Palmitic acid_HAp has the greatest band gap of 5.58 eV, indicating that long-chain fatty acid functionalization significantly affects the electrical structure of the HAp material.

**Table 3:** Measured band gap of synthesized HAp

Sample name	*E*_g_ (eV)
Pure_HAp	5.25
Doped_HAp	5.45
Urea_HAp	5.13
Naphthalene_Hap	5.32
Palmitic acid_HAp	5.58

### Surface morphology study

**Fig. 11:**
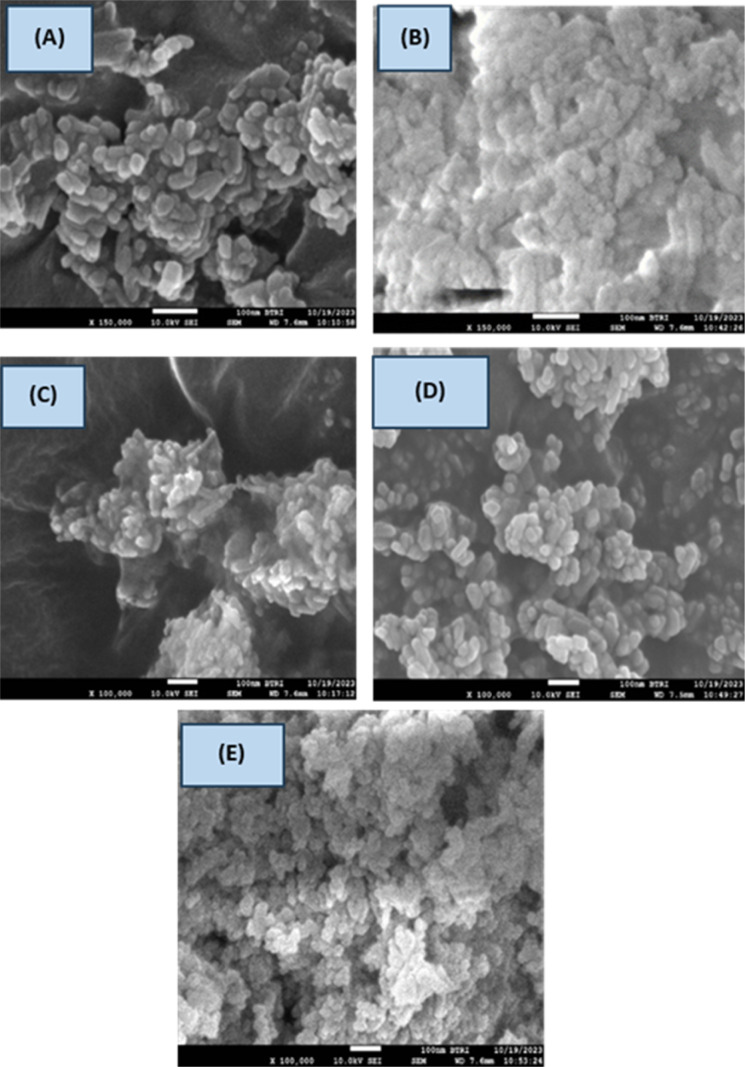
Surface morphology of the sample (A) Pure HAp, (B) Doped_HAp, (C) Urea_HAP, (D) Naphthalene_HAp, (E) Palmitic acid_HAp.

The surface morphology of the produced HAp was analyzed from the SEM images of the hydrothermally synthesized HAp. It is prominently visible that the synthesized HAp showed distinguishing shapes. Organic modifiers such as urea, naphthalene, and palmitic played a crucial role in altering the morphology and crystal structure of the synthesized HAp. Urea functions as a mineralizer, facilitating the formation of HAp nanorods and nanobundles.^13^ But in the case of HAp, no such strong effects were observed; rather, the formation of small aggregates was found, which is visualized in Fig. 11(C). The surface of HAp nanoparticles was influenced by urea, which promotes the growth of smaller, less crystalline structures, while palmitic acid, as a surfactant, restricted the size distribution and growth.^54^ Naphthalene formed pore-creating structures, resulting in high surface area and porosity in HAp, which helped control the particle size and prevent excessive aggregation.^55^ The porous nature of these HAp aggregates was likely due to the presence of the naphthalene template. Pure HAp, urea_HAp, and naphthalene_HAp possess monodispersed nanorod shapes with a length of 53.44, 38.74, and 53.23 nm, respectively.^56^ Apart from that, several associated particles with no distinct geometric forms were observed for doped HAp and palmitic acid HAp with crystallite sizes 30.89 and 50.79 nm, respectively. The particle sizes determining using SEM using 10–20 population are shown in Table 4. Normally, freeze drying is chosen for the drying of nanomaterials, but in this case, 105 °C was the temperature, which may be relatively higher for the formation of nano-aggregates. The synthesized samples exhibited particle aggregation, possibly due to higher temperatures, causing solvent fluctuations and Brownian motion, resulting in enhanced nuclei collisions and agglomeration, forming larger nanoparticles.^57^ The surface charge of the nanoparticles affects their aggregation, with larger particles exhibiting stronger repulsive interactions and smaller particles having lower charges, thus facilitating easier adhesion and agglomeration.^58^

**Table 4:** Crystallite size of synthesized HAp

Sample name	Coefficient of variation (CV)	Average particle size (nm)	Marginal error
Pure HAp	39.265	53.442	10.494
Dope HAp	34.251	30.890	9.887
Urea HAp	35.57	38.745	10.268
Naphthalene HAp	27.604	53.236	7.127
Palmitic acid HAp	30.67600	50.798	10.845

## Rietveld refinement

The quantitative analysis of hydrothermally synthesized HAp was conducted using Profex (5.2.4) software to obtain the quantitative phase percentages of hydroxyapatite using the Rietveld refinement technique. The phase percentage was estimated using 6500 points measuring angles (2*θ* = 5–70°, step of 0.01). The hydrothermally synthesized HAp shows different phase percentages, which are listed in Table 5.

**Table 5:** Different phase estimation *via* the Rietveld refinement method

Samples	HAp phase (%)	Alpha TCP (%)	Beta TCP (%)	*R*_wp_	*R*_exp_	*χ* ^2^	GoF
Pure HAp	93.35	2.80	3.86	13.54	17.24	0.62	0.79
Dope HAp	82.15	8.49	9.36	15.45	17.46	0.78	0.88
Naphthalene HAp	96.63	1.43	1.94	12.96	17.14	0.57	0.76
Palmitic acid HAp	87.40	5.78	6.85	14.37	16.95	0.72	0.85

## Conclusion

This study successfully synthesized hydroxyapatite (HAp) through a hydrothermal process, which was analyzed using various techniques including XRD, FTIR, SEM, and UV-vis spectroscopy. The synthesized HAp possesses an accurate crystallographic parameter, which is an indication of the formation of the HAp nanocrystal. This study proposed that the crystalline structure of HAp can be altered through the hydrothermal reaction using organic modifiers such as urea, naphthalene, as well as palmitic acid. The synthesized HAp exhibits morphology like monodispersed nanorods along with varying aggregation. The result obtained from the XRD model revealed that the synthesized product has nano-sized (<100 nm), retaining energy density, strain, and internal stress. This analysis utilized the Scherrer plot, different forms of Williamson–Hall plot, Sahadat-Scherrer's method, size–strain plot, and Halder–Wagner method. Furthermore, the average nanocrystal size was determined and confirmed from the SEM image, which validated the result obtained from XRD analysis. After analyzing all these examinations, it has been demonstrated that the Williamson–Hall approach delivers the best result for the calculation of elastic properties as well as crystallite size.

## Data availability

Data will be made available on request.

## Author contributions

Md. Kawsar synthesized the hydroxyapatites and wrote the draft and original manuscript. Md. Sahadat Hossain conceived and designed the experiment and analyzed the data. Sumaya Tabassum executed the bandgap energy. Dipa Islam executed the SEM analysis. Newaz Mohammed Bahadur and Samina Ahmed supervised the findings of this work. Samina Ahmed supervised the overall work and manage the required facilities.

## Conflicts of interest

There are no conflicts to declare.
